# Supplementary material for: Dose-related association between radiation exposure from computed tomography (CT) scans during trauma hospitalizations and subsequent risk of developing new-onset cancers
Source: Commun Med (Lond). 2026 Jan 5;6:89. doi: 10.1038/s43856-025-01354-z (PMC12881511; doi:10.1038/s43856-025-01354-z)
Supplement: Supplementary file 2 — Supplementary Materials [file 43856_2025_1354_MOESM2_ESM.pdf]

Supplementary Materials

	Page
**Table S1.** Differences in patient characteristics between smokers in the main dataset (n=1,251) and those in the imputed datasets.	2
**Table S2.** List of cancer-related deaths following the index trauma admission (n=21 out of 2,659 patients).	3
**Table S3.** Cox proportional hazards regression was used to assess the associations between clinical covariates and the risk of cancer-related deaths following trauma admission. The analysis included 170 cancer-related deaths among 15,352 patients, after applying multiple imputation for missing smoking status.	4
**Table S4.** Cox proportional hazards regression was used to assess the associations between clinical covariates and the risk of developing any new cancer cases following trauma admission. The analysis included 591 new-onset cancer cases among 15,352 patients, after applying multiple imputation for missing smoking status.	5
**Table S5.** Cox proportional hazards regression was used to assess the associations between clinical covariates and the risk of developing the first type of cancer following the injury, stratified by the anatomical site of injury — presumed to be the region most frequently scanned. The analysis included a total of 591 new-onset cancer cases among 15,352 patients, after applying multiple imputation for missing smoking status. Radiation dose exposure was dichotomized.	6-7
**Table S6.** Cox proportional hazards regression was used to assess the associations between clinical covariates and the risk of developing the first type of cancer following the injury, stratified by the anatomical site of injury — presumed to be the region most frequently scanned. The analysis included a total of 591 new-onset cancer cases among 15,352 patients, after applying multiple imputation. Radiation dose exposure was modelled as a continuous predictor.	8-9
**Figure S1.** Differences in the cumulative incidence of cancer-related deaths following trauma admission were assessed (a) by dichotomizing patients based on exposure to radiation dose-length product < or > 5,000 mGy*cm, and (b) by categorizing patients according to radiation dose exposure ranges, with adjustment for covariates listed in Table S3.	10
**Figure S2.** Difference in the cumulative incidence of lymphoma or leukemia following trauma admission, adjusted for covariates listed in Table S5.	11
**Figure S3.** Difference in the cumulative incidence of head or neck cancers following trauma admission, adjusted for covariates listed in Table S5.	12
**Supplementary Notes:** Description of the variables contained in the dataset.	13-15

Table S1. Differences in patient characteristics between smokers in the main dataset (n=1,251) and those in the imputed datasets.

Variable	Original dataset	Imputed dataset
Mean age, years (SD)	37.4 (15.5)	43.2 (20.8)
Male, no. (%)	995 (79.5)	19,095 (69.8)
Mean ISS (SD)	20.2 (7.1)	10.9 (8.8)
Charlson comorbidity index	0.10 (0.41)	0.14 (0.52)
Length of hospital stay, days (SD)	11.6 (16.5)	6.5 (11.4)
Survival time until censor date, years (SD)	6.3 (3.0)	6.6 (3.1)
Died before censor date, no. (%)	163 (13.0)	3,942 (14.4)

SD, standard deviation. ISS< Injury Severity Score.

Table S2. List of cancer-related deaths following the index trauma admission (n=21 out of 2,659 patients).

Cause of death	Time from the index trauma admission among those without documented cancer diagnosis within 5 years prior to trauma admission (years)
LARYNGEAL CANCER	2.9
GLIOBLASTOMA MULTIFORME*	7.0
LUNG CANCER	7.3
LUNG CANCER	5.5
KIDNEY CANCER	2.4
GLIOBLASTOMA MULTIFORME	1.0
NON-HODGKIN LYMPHOMA*	5.4
PROSTATE CANCER	4.4
RECTUM CANCER*	1.3
MALIGNANT CONNECTIVE AND SOFT TISSUE NEOPLASM	4.7
PLEURAL MESOTHELIOMA	9.3
PROSTATE CANCER	1.3
MULITPLE MYELOMA	3.7
PANCREAS CANCER	3.1
LUNG CANCER	4.8
PANCREAS CANCER	6.5
PANCREAS CANCER	5.1
GLIOBLASTOMA MULTIFORME	2.4
OESOPHAGUS CANCER*	7.1
MALIGNANT MELANOMA*	6.5
LUNG CANCER	2.2

*Those who had received dose-length-product radiation exposure greater than 5,000 mGy*cm from CT scans during the index trauma admission.

Table S3. Cox proportional hazards regression was used to assess the associations between clinical covariates and the risk of cancer-related deaths following trauma admission. The analysis included 170 cancer-related deaths among 15,352 patients, after applying multiple imputation for missing smoking status.

**Analyzing radiation exposure as a categorical variable**	**Hazard ratio (95% CI)**	**P-value**
Age	1.08 (1.07-1.08)<br>per year increment	*0.001*
Diabetes mellitus	1.29 (1.05-1.59)	*0.015*
Smoker (yes vs no)	1.13 (0.98-1.30)	0.085
Alcohol user (yes vs no)	1.01 (0.77-1.34)	0.924
Ischemic heart disease	0.97 (0.66-1.41)	0.857
Congestive heart failure	0.58 (0.38-0.88)	*0.011*
Chronic pulmonary disease	1.15 (0.83 -1.59)	0.394
CT scan radiation dose-length-product >5,000 mGy*cm during index trauma hospitalization	2.08 (1.47-2.95)	*0.001*
**Analyzing radiation exposure as a continuous variable**	**Hazard ratio (95% CI)**	**P-value**
Age	1.08 (1.07-1.08)<br>per year increment	*0.001*
Diabetes mellitus	1.30 (1.06-1.60)	*0.014*
Smoker (yes vs no)	1.13 (0.99-1.30)	0.079
Alcohol user (yes vs no)	1.07 (0.81-1.41)	0.471
Ischemic heart disease	0.96 (0.66-1.41)	0.834
Congestive heart failure	0.58 (0.38-0.88)	*0.010*
Chronic pulmonary disease	1.14 (0.83 -1.58)	0.425
CT scan radiation exposure during index trauma hospitalization	1.03 (0.99-1.08)<br>per 1,000 mGy*cm increment	0.184

Table S4. Cox proportional hazards regression was used to assess the associations between clinical covariates and the risk of developing any new cancer cases following trauma admission. The analysis included 591 new-onset cancer cases among 15,352 patients, after applying multiple imputation for missing smoking status.

**Analyzing radiation exposure as a categorical variable**	**Hazard ratio (95% CI)**	**P-value**
Age	1.06 (1.06-1.06)<br>per year increment	*0.001*
Diabetes mellitus	1.54 (1.39-1.70)	*0.001*
Smoker (yes vs no)	0.98 (0.92-1.06)	0.636
Alcohol user (yes vs no)	1.29 (1.15-1.45)	*0.001*
Ischemic heart disease	0.73 (0.59-0.90)	*0.004*
Congestive heart failure	0.91 (0.75-1.10)	0.331
Chronic pulmonary disease	1.32 (1.13-1.55)	*0.001*
CT scan radiation dose-length-product >5,000 mGy*cm during index trauma hospitalization	1.37 (1.14-1.65)	*0.001*
**Analyzing radiation exposure as a continuous variable**	**Hazard ratio (95% CI)**	**P-value**
Age	1.06 (1.06-1.06)<br>per year increment	*0.001*
Diabetes mellitus	1.53 (1.38-1.70)	*0.001*
Smoker (yes vs no)	0.99 (0.92-1.06)	0.690
Alcohol user (yes vs no)	1.27 (1.12-1.43)	*0.001*
Ischemic heart disease	0.73 (0.59-0.90)	*0.004*
Congestive heart failure	0.91 (0.75-1.10)	0.328
Chronic pulmonary disease	1.33 (1.14-1.55)	*0.001*
CT scan radiation exposure during index trauma hospitalization	1.05 (1.02-1.08)<br>per 1,000 mGy*cm increment	*0.001*

Table S5. Cox proportional hazards regression was used to assess the associations between clinical covariates and the risk of developing *the first type of cancer* following the injury, *stratified by the anatomical site of injury* — presumed to be the region most frequently scanned. The analysis included a total of 591 new-onset cancer cases among 15,352 patients, after applying multiple imputation for missing smoking status. Radiation dose exposure was dichotomized.

	**Hazard ratio (95% CI)**	**P-value**
**Injury to any region**	**Lymphoma or leukemia (n=21)**	
Age	1.04 (1.03-1.05) per year increment	*0.001*
Diabetes mellitus	1.74 (0.99-3.03)	0.051
Smoker (yes vs no)	0.63 (0.42-0.94)	*0.024*
Alcohol user (yes vs no)	0.97 (0.521.81)	0.925
Ischemic heart disease	0 (0-0)	0.957
Congestive heart failure	2.36 (0.94-5.91)	0.067
Chronic pulmonary disease	0 (0-0)	0.960
CT scan radiation dose-length-product >5,000 mGy*cm during index trauma hospitalization	3.75 (2.01-6.99)	*0.001*
**Thoracic injury**	**Thoracic region cancers (n=107)**	
Age	1.06 (1.06-1.07) per year increment	*0.001*
Diabetes mellitus	1.61 (1.27-2.03)	*0.001*
Smoker (yes vs no)	1.22 (1.04-1.44)	*0.014*
Alcohol user (yes vs no)	1.23 (0.93-1.64)	0.149
Ischemic heart disease	0.96 (0.60-1.53)	0.861
Congestive heart failure	0.50 (0.29-0.86)	*0.012*
Chronic pulmonary disease	1.45 (1.02-2.05)	*0.037*
CT scan radiation dose-length-product >5,000 mGy*cm during index trauma hospitalization	0 (0-0)	0.909
**Abdominal injury**	**Gastrointestinal or pelvic cancers (n=162)**	
Age	1.05 (1.05-1.06) per year increment	*0.001*
Diabetes mellitus	1.31 (1.06-1.61)	*0.014*
Smoker (yes vs no)	0.93 (0.81-1.07)	0.304
Alcohol user (yes vs no)	1.07 (0.84-1.36)	0.577
Ischemic heart disease	0.70 (0.45-1.09)	0.702
Congestive heart failure	0.74 (0.49-1.11)	0.145
Chronic pulmonary disease	1.55 (1.16-2.08)	*0.003*
CT scan radiation dose-length-product >5,000 mGy*cm during index trauma hospitalization	0 (0-0)	0.853

**Head and neck injury**	**Head or Neck cancers (n=21)**	
Age	1.02 (1.01-1.03)<br>per year increment	*0.001*
Diabetes mellitus	1.66 (0.85-3.25)	0.140
Smoker (yes vs no)	1.22 (0.85-1.76)	0.278
Alcohol user (yes vs no)	1.36 (0.81-2.28)	0.249
Ischemic heart disease	3.02 (1.21-7.55)	*0.018*
Congestive heart failure	0 (0-0)	*0.961*
Chronic pulmonary disease	0 (0-0)	0.958
CT scan radiation dose-length-product >5,000 mGy*cm during index trauma hospitalization	2.64 (1.14-6.11)	*0.023*
**Injury to all regions**	**Metastatic cancer (n=152)**	
Age	1.06 (1.06-1.07)<br>per year increment	*0.001*
Diabetes mellitus	1.04 (0.83-1.30)	0.748
Smoker (yes vs no)	1.16 (1.01-1.33)	*0.036*
Alcohol user (yes vs no)	1.09 (0.85-1.40)	0.498
Ischemic heart disease	0.78 (0.51-1.18)	0.239
Congestive heart failure	0.79 (0.54-1.16)	0.226
Chronic pulmonary disease	1.67 (1.26-2.20)	*0.001*
CT scan radiation dose-length-product >5,000 mGy*cm during index trauma hospitalization	1.40 (0.96-2.05)	0.083

Table S6. Cox proportional hazards regression was used to assess the associations between clinical covariates and the risk of developing the *first type of cancer* following the injury, *stratified by the anatomical site of injury* — presumed to be the region most frequently scanned. The analysis included a total of 591 new-onset cancer cases among 15,352 patients, after applying multiple imputation. Radiation dose exposure was modelled as a continuous predictor.

	**Hazard ratio (95% CI)**	**P-value**
**Injury to any region**	**Lymphoma or leukemia (n=21)**	
Age	1.04 (1.03-1.05) per year increment	*0.001*
Diabetes mellitus	1.78 (1.02-3.11)	*0.042*
Smoker (yes vs no)	0.63 (0.42-0.94)	*0.027*
Alcohol user (yes vs no)	1.06 (0.57-1.98)	0.852
Ischemic heart disease	0 (0-0)	0.955
Congestive heart failure	2.28 (0.91-5.70)	0.079
Chronic pulmonary disease	0 (0-0)	0.959
CT scan radiation exposure during index trauma hospitalization	1.09 (0.99-1.20) per 1,000 mGy*cm increment	0.054
**Thoracic injury**	**Thoracic region cancers (n=107)**	
Age	1.06 (1.06-1.07) per year increment	*0.001*
Diabetes mellitus	1.61 (1.27-2.03)	*0.001*
Smoker (yes vs no)	1.22 (1.04-1.44)	*0.014*
Alcohol user (yes vs no)	1.23 (0.93-1.64)	0.150
Ischemic heart disease	0.96 (0.60-1.53)	0.855
Congestive heart failure	0.50 (0.29-0.86)	*0.013*
Chronic pulmonary disease	1.45 (1.02-2.06)	*0.036*
CT scan radiation exposure during index trauma hospitalization	0.94 (0.81-1.10) per 1,000 mGy*cm increment	0.439
**Abdominal injury**	**Gastrointestinal or pelvic cancers (n=162)**	
Age	1.05 (1.05-1.06) per year increment	*0.001*
Diabetes mellitus	1.30 (1.05-1.60)	*0.016*
Smoker (yes vs no)	0.93 (0.81-1.07)	0.314
Alcohol user (yes vs no)	1.06 (0.83-1.34)	0.656
Ischemic heart disease	0.71 (0.45-1.10)	0.119
Congestive heart failure	0.74 (0.49-1.12)	0.149
Chronic pulmonary disease	1.56 (1.16-2.08)	*0.003*
CT scan radiation exposure during index trauma hospitalization	1.02 (0.94-1.11)	0.625

	per 1,000 mGy*cm increment	
**Head and neck injury**	**Head or Neck cancers (n=21)**	
Age	1.02 (1.01-1.03) per year increment	*0.001*
Diabetes mellitus	1.69 (0.86-3.30)	0.128
Smoker (yes vs no)	1.23 (0.86-1.77)	0.255
Alcohol user (yes vs no)	1.28 (0.76-2.14)	0.352
Ischemic heart disease	2.97 (1.19-7.44)	*0.020*
Congestive heart failure	0 (0-0)	0.961
Chronic pulmonary disease	0 (0-0)	0.960
CT scan radiation exposure during index trauma hospitalization	1.15 (1.07-1.24) per 1,000 mGy*cm increment	*0.001*
**Injury to all regions**	**Metastatic cancer (n=152)**	
Age	1.06 (1.06-1.07) per year increment	*0.001*
Diabetes mellitus	1.04 (0.83-1.30)	0.753
Smoker (yes vs no)	1.16 (1.01-1.33)	*0.032*
Alcohol user (yes vs no)	1.06 (0.82-1.37)	0.649
Ischemic heart disease	0.78 (0.51-1.18)	0.235
Congestive heart failure	0.79 (0.54-1.16)	0.227
Chronic pulmonary disease	1.67 (1.26-2.21)	*0.001*
CT scan radiation exposure during index trauma hospitalization	1.04 (1.01-1.08) per 1,000 mGy*cm increment	*0.046*

Figure S1. Differences in the cumulative incidence of cancer-related deaths following trauma admission were assessed (a) by dichotomizing patients based on exposure to radiation dose-length product < or > 5,000 mGy*cm, and (b) by categorizing patients according to radiation dose exposure ranges, with adjustment for covariates listed in Table S3.

(a)

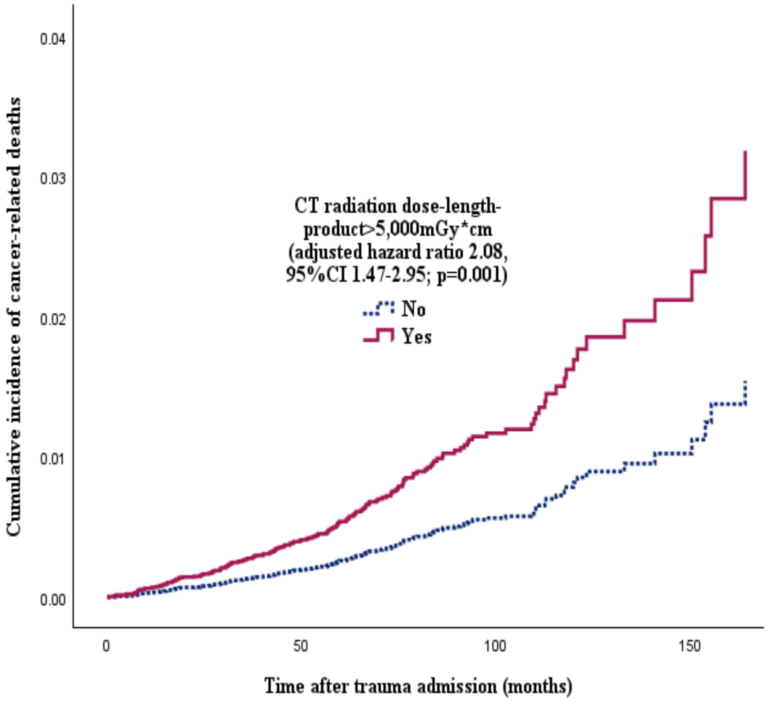

(b)

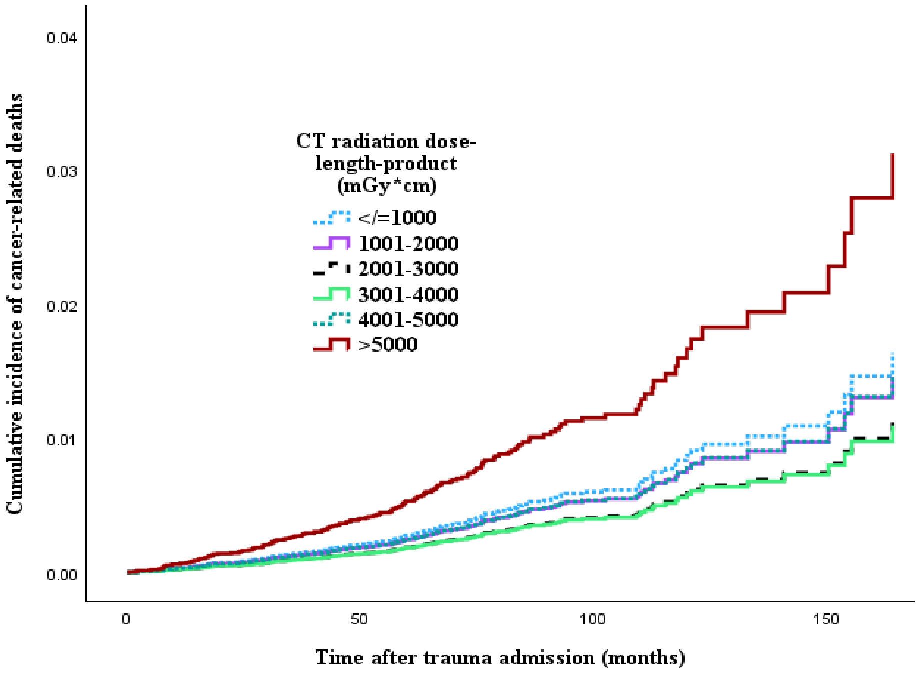

Figure S2. Difference in the cumulative incidence of lymphoma or leukemia following trauma admission, adjusted for covariates listed in Table S5.

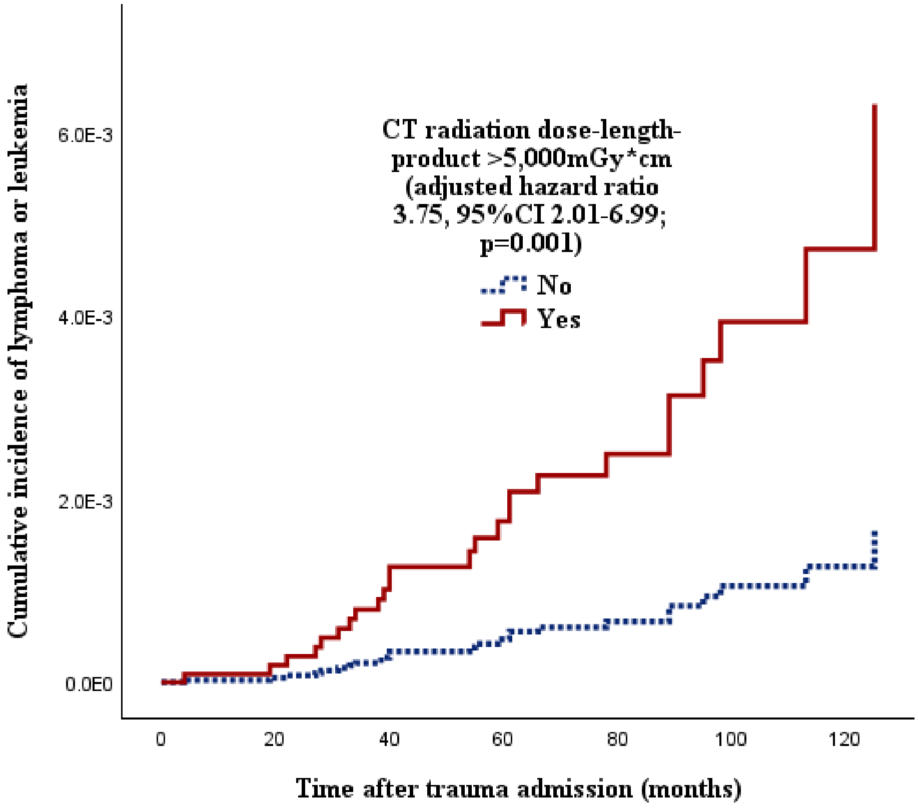

Figure S3. Difference in the cumulative incidence of head or neck cancers following trauma admission, adjusted for covariates listed in Table S5.

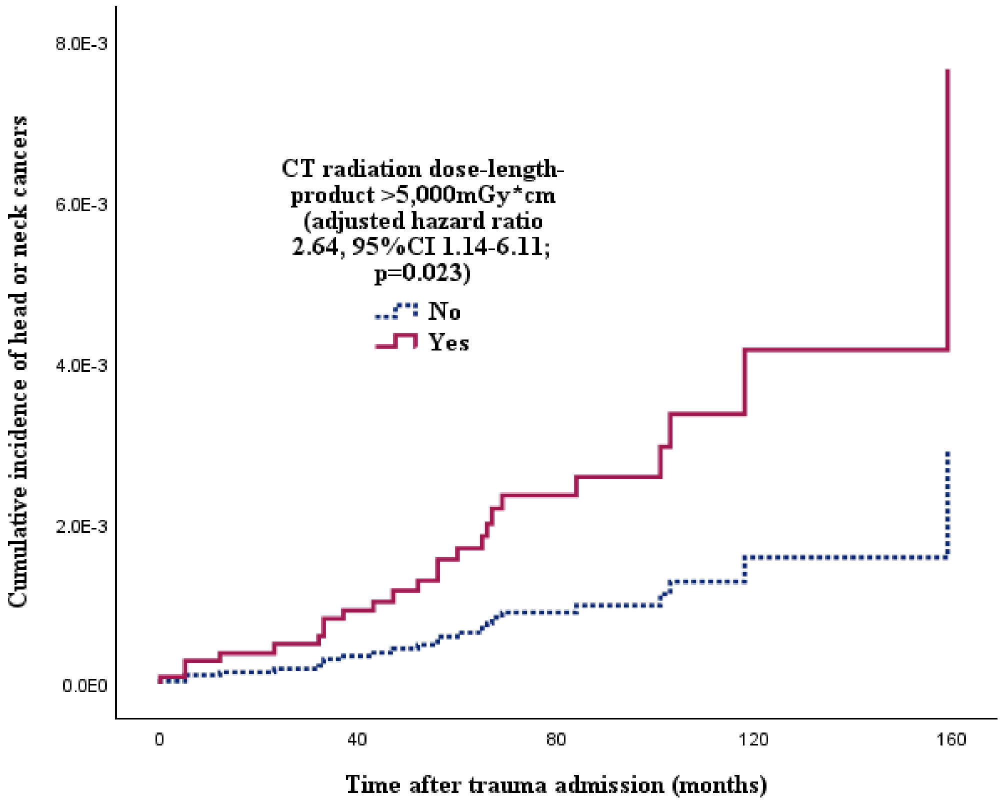

Supplementary Notes: Description of the variables contained in the dataset

Note that age, year of birth, sex, ethnicity, and location of injury of the study patients have been removed to ensure patients remain unidentifiable

1. Death due to cancer, censored those occurred within 3 years (1 = yes)
2. Death due to cancer (1 = yes)
3. Cancer cause (of death): free text
4. Time to death (in years)
5. Adm_date (for trauma admission)
6. Brain_cancer (1=yes)
7. Head_neck_cancers (1=yes)
8. Cancer_type (or region involved)
9. Cancer_date (date of the diagnosis of cancer)
10. New_onset_Cancer_posttrauma (1= yes)
11. Length_of_stay (in hospital in days)
12. ICU_days
13. ICU_hours
14. Neck_cervical_spine_injury_adm (1= yes)
15. Thoracic_injury_adm (1= yes)
16. Abdo_injury_including_lumbar_spine_inj_adm (1= yes)
17. UL_injury_adm (1= yes)
18. LL_injury_adm (1= yes)
19. Burns_adm (1= yes)
20. FB_penetration_natural_orifice_adm (1= yes)
21. Unspecified_injury_adm (1= yes)
22. Multi_region_injury_adm (1= yes)
23. TBI_adm (1= yes)
24. Mechanism_of_injury
25. Principal_diagnosis (ICD-10 coding)
26. Co_diagnosis and other diagnoses (ICD-10 coding)
27. Principal_procedure (ICD-10 coding)
28. Advantage_LGA_score_2016Disadvantage_LGA_Score_2016
29. Economic_Resources_LGA_Score_2016
30. National_edu_occup_LGA_score_2016
31. Charlson comorbidities (individual components, 1=yes)
32. Cancers_after_trauma (1= yes)
33. Weight (in kg)
34. Height (in metres)
35. Therapeutic_drugs (anticoagulants)
36. Activation_criteria_met
37. TRISS_initial
38. TRISS_final
39. Cardiac_arrest
40. Hypotension
41. Vomiting
42. Airway_compromised
43. External_bleeding

44. Other_conditions
45. Alcohol_level
46. Alcohol_within_12hrs
47. Illicit_drugs_within_12hrs
48. Cause_work_related
49. Location
50. Location_type
51. Safety_device
52. Safety_device_type
53. Holiday_related
54. Trauma_against
55. Final_observation_at Final_DBP
56. death_year
57. death_age
58. Death_primary_ICD_code
59. Cause_death_text1
60. Cause_death_text2
61. Cause_death_text3
62. Cause_death_procedure_related
63. Death_procedures
64. CountXray
65. CountCT
66. CountValidDoseScreen
67. TotalReportedDLP
68. TotalCalculatededDLP
69. Date_death
70. Discharge_date
71. Censor_or_death_date
72. Date_cancer_censor
73. Death (during the whole follow-up period)
74. Survival_days
75. Survival_months
76. Survival_years
77. Recurrent_trauma_hospitalisation_number
78. Recurrent_trauma_yes_no
79. ISS
80. Mortality_30D (1= death within 30 days)
81. Mortality_1Y (1= death within 1 year)
82. Smoker (1= yes)
83. Drinker (1=yes)
84. Major_trauma (1= ISS>15)
85. CT_Y_N (1= CT scan was done)
86. Measured_radiation_dose_DLP (in mGy*cm)
87. Estimated_dose_mSV
88. DM (1=diabetes mellitus)
89. Radiation_dose_1000 (numerical value per 1000 mGy*cm)

90. More_than_5000CTdose (1= more than 5000 mGy*cm)
91. Time_to_cancer_censor (time to cancer diagnosis in years)
92. Time_censored_death_cancer (time to cancer diagnosis or death from all causes)
93. Below_WBCT_dose (1= unlikely to have received a standard whole body CT scan)
