# Supplementary material for: Dose-related association between radiation exposure from computed tomography (CT) scans during trauma hospitalizations and subsequent risk of developing new-onset cancers
Source: Commun Med (Lond). 2026 Jan 5;6:89. doi: 10.1038/s43856-025-01354-z (PMC12881511; doi:10.1038/s43856-025-01354-z)
Supplement: Supplementary file 3 — Description of Additional Supplementary files [file 43856_2025_1354_MOESM3_ESM.pdf]

Description of Additional Supplementary Files

File name: Supplementary Data 1

Description: A deidentified version of the dataset. Note that age, year of birth, sex, ethnicity, and location of injury of the study patients have been removed to ensure patients remain unidentifiable.
